# Development the method of pipeline bending strain measurement based on microelectromechanical systems inertial measurement unit

**DOI:** 10.1177/0036850420925231

**Published:** 2020-06-15

**Authors:** Rui Li, Zhensheng Wang, Pengchao Chen

**Affiliations:** 1 School of Automation Science and Electrical Engineering, Beihang University, Beijing, China; 2 Petrochina Pipeline Company, Langfang, China

**Keywords:** In-line inspection, pipeline bending strain, microelectromechanical systems inertia navigation, extended Kalman filter, wavelet analysis

## Abstract

With the development of pipeline construction, the additional stress and strain becomes the key factor to induce the damage for oil and gas pipeline. The in-line inspection of pipeline bending strain which is based on high-end tactical-grade inertial measurement unit has become routine practice for the oil and gas pipelines over recent years. However, these accurate inertial measurement units are large size and high cost limit to use in small diameter pipelines of bending strain inspection. Microelectromechanical systems–based inertial navigation has been applied to mapping the centerline of the small size pipeline, and the accurate trajectory and attitude information become key factors to calculate the bending strain of pipelines. This article proposed a method not only to calculate the pipeline bending strain but also to improve the accuracy for the bending strain based on the wavelet analysis. Tests show that this method can be effectively used in the calculation and optimization of the bending strain, and it will increase the accuracy to within 19.1% of the actual bending strain.

## Introduction

With the rapid development of economy and society, industries such as oil, gas, water, sewage, and chemical industry are increasingly relying on large underground pipeline transportation services,^1^ and the efficiency and reliability of these services are guaranteed by pipeline inspection, by monitoring and subsequent maintenance. Especially, for the long-distance oil and gas pipeline, it may be induced by landside, displacement, and deformation because of geologic hazards.^2^ The bending strain will be produced due to these displacement and deformation. Therefore, the bending strain of the pipeline seriously affects the structural integrity and operational safety of the pipeline, especially when there are serious defects with the bending strain, which are more likely to cause pipeline failure and rupture.^3^ Therefore, the effective method for inspection of the pipeline bending strain becomes one of the key techniques to avoid the cracking of the pipeline due to the severe bending strain caused by the external stress.

At present, for the big size (more than 10″) pipeline, we generally use a pipeline inspection tool which installs a high-precision tactical-level inertial measurement unit (IMU) to measure the geographic trajectory of underground pipelines.^4,5^ Then the pipeline bending strain is calculated by the accurate trajectory of centerline and measured attitude of pipeline. However, the tactical-level IMU is too large to be used in small-diameter pipelines, and its cost is too high. Therefore, for small-size pipeline inspection, small and inexpensive microelectromechanical systems (MEMS) IMU have to be considered to install in the in-inspection tool. However, the MEMS IMU is characterized by large uncertainties and high noise such as bias, scale factor, and non-orthogonality.^6^ Without other correction information, the position drift error of MEMS IMU is not suppressed, and its measurement accuracy drops sharply with time, which cannot be applied in practical inspection.

**Figure 1.**
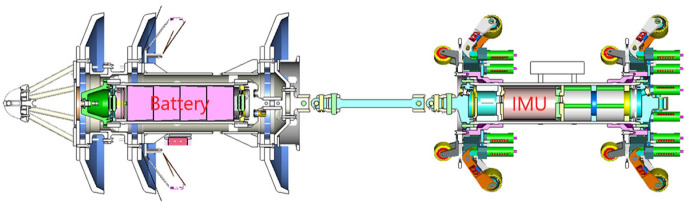
IMU in-line inspection tool.

Nowadays, different companies and organizations have proposed method of combined navigation between MEMS IMU and other auxiliary information to improve the accuracy of navigation and positioning.^7,8^ A typical IMU in-line inspection tool (IIT) is composed of a vehicle, MEMS IMU, odometer, and above-ground marker (AGM) system, as shown in Figure 1. The odometer is capable of measuring the distance traveled by the IIT and can be converted to forward speed to suppress the accumulation of navigation positioning errors. AGM is a ground marker with known coordinates that provide position correction information for the IIT. The non-integrity constraint is a kind of virtual observation value proposed according to the motion of the IIT and provides the speed auxiliary information in the carrier coordinate system.

Due to the large error of the MEMS inertial navigation system, it is easy to receive external disturbances during operation such as the girth weld and vibration. The trajectory and attitude information of the IIT is disturbed, which affects the accuracy of the inspection. In this article, an in-line inspection method and algorithm for pipeline centerline trajectory and bending strain of small-size pipeline will be proposed. Based on this method, to improve the inspection precision of bending strain, a new optimization based on wavelet analysis will be proposed. The effectiveness and accuracy of the method will be proved through the practice field tests.

## Algorithm for pipeline centerline trajectory based on multi-sensors data fusion

The IMU IIT can collect data including angular velocity and acceleration in three directions during the inspection. The navigation information (position, velocity, and attitude) is obtained through inertial navigation such as projection and integration.^9^ Due to the influence of MEMS IMU errors of position, velocity and attitude estimated by inertial navigation system will accumulate rapidly, which seriously affects the engineering application. It is impossible for the IMU IIT underground pipeline to use global positioning system (GPS) information to assist in suppressing the error accumulation of the inertial navigation. Other ancillary information, such as odometers, AGM, and motion constraints, can be used as an alternative aid to effectively suppress the accumulation of errors.

Kalman filtering has been used to solve the problem for high-end tactical-grade IMU in-line inspection of state or parameter estimation of stochastic linear discrete systems. However, due to the limitation of size of the pipeline, small-size pipelines can only use the MEMS IMU for pipeline centerline measurement.^10^ The Kalman filtering is not applicable to the state or parameter estimation of the system for the MEMS IMU because of its continuous nonlinearity. For this reason, the extended Kalman filter (EKF) can be used to linearize the error state of the nonlinear continuous system to achieve optimal estimation.^11,12^

The error state of the inertial navigation system is designed as follows with position error $\delta p^{n}$ , velocity error $\delta v^{n}$ , attitude error ψ , gyro bias $\delta b_{g}$ , acceleration deviation $\delta b_{a}$

(1) $$\delta x={\left[\delta p^{n}\;\delta v^{n}\;\psi \;\delta b_{g}\;\delta b_{a}\right]}^{T}$$

where $\delta p^{n}=[\delta p_{N}\;\delta p_{E}\;\delta p_{D}]$ is the north, east, and vertical position error. $\delta v^{n}=[\delta v_{N}\;\delta v_{E}\;\delta v_{D}]$ is the north, east, and vertical speed error. $\psi =[\psi_{r}\;\psi_{p}\;\psi_{y}]$ is the roll, pitch, and heading error. $\delta b_{g}$ and $\delta b_{a}$ are gyro offset error and accelerometer offset error, respectively.

The error state model of IMU after discrete linearization can be expressed as

(2) $$\delta x_{k+1}=\Phi_{k}\delta x_{k}+G_{k}w_{k}$$

where $\delta x_{k+1}$ is the state vector, $\Phi_{k}$ is the state transition matrix, $G_{k}$ is the system noise drive matrix, and $w_{k}$ is the state noise vector.

Based on the above dynamic system state model, an observation model is also needed to provide correction information for the system state vector. The observation model $\delta z_{k}$ proposed is based on the AGM and odometer. The linearized observation error model can be expressed as

(3) $$\delta z_{k}=H\delta x_{k}+v_{k}$$

where H is the matrix of observation coefficient, and $v_{k}$ is the observed noise. The observation noise for position and odometer are considered to be white noise and independent of each other. The position from AGM of observation equation error can be expressed as

(4) $$\delta z_{k}=\left[\begin{array}{c} 1\;0\;0\\ 0\;1\;0\\ 0\;0\;1 \end{array}\right]\left[\begin{array}{c} \delta r_{N}\\ \delta r_{E}\\ \delta r_{D} \end{array}\right]+\left[\begin{array}{c} w_{N}\\ w_{E}\\ w_{D} \end{array}\right]$$

The observation equation of the speed error is expressed as

(5) $$\delta z_{v}=C_{b}^{v}C_{n}^{b}\delta v_{\mathrm{IMU}}^{n}-C_{b}^{v}C_{n}^{b}\left(v_{\mathrm{IMU}}^{n}\times \right)\psi -e_{v}$$

where $C_{b}^{v}$ is the transformation matrix of the carrier coordinate system to the IIT coordinate system, $C_{n}^{b}$ is the transformation matrix of the navigation coordinate system to the carrier coordinate system, and $e_{v}$ is the observing noise for speed.

The error estimated by the EKF is the latest time estimate of the system state vector, and the filter works is a closed loop with error feedback correction. Therefore, when the system error state vector completes the observation update each time, it is used to correct the navigation state and parameters and reset the error state vector to zero.

The state update equation is expressed in discrete form as follows

(6) $$\delta x=K_{k}\left(Z_{k}-H_{k}x_{k|k-1}\right)$$

where *K_k_* is the gain matrix. The corresponding error covariance matrix as follows

(7) $$P_{k|k-1}=A_{k,k-1}P_{k-1}A_{k,k-1}^{T}+Q_{k}$$

where P is the covariance matrix of the error state, $A_{k,k-1}$ is the state transition matrix, and $Q_{k}$ is the covariance matrix of the system noise. The covariance matrix of the EKF update estimated state error is as follows

(8) $$K_{k}=P_{k|k-1}H_{k}^{T}{\left(H_{k}P_{k|k-1}H_{k}^{T}+R_{k}\right)}^{-1}$$

(9) $$P_{k}=\left(I-K_{k}H_{k}\right)P_{k|k-1}{\left(I-K_{k}H_{k}\right)}^{T}+K_{k}R_{k}K_{k}^{T}$$

where $R_{k}$ is covariance matrix for the observation.

## Calculation and optimization for pipeline bending strain

### The calculation method of the pipeline bending strain

The pipeline bending strain is due to the lateral additional loadings. If the pipeline is affected by such as earthquakes, frozen and frozen areas, natural disasters floods, and human excavation disturbances, it will cause large pipeline strain and displacement, which will threaten the safety of the pipeline. The IIT can accurately measure the centerline position of the pipeline. Generally, within the elastic deformation range of the pipe material, the bending strain is proportional to the curvature of the pipeline. Therefore, the curvature of the pipeline is accurately calculated by the position information of the centerline, and the pipeline bending strain can be calculated.

The pipeline bending strain can be expressed as follows

(10) $$\varepsilon =\frac{\mathrm{kD}}{2}$$

where ε is the pipeline bending strain, and *D* is the pipeline diameter. The total curvature of the centerline of a pipe is described at each point along the pipeline by the curvature vector. In order to calculate the pipeline curvature, the centerline of a pipe is considered as a 3D parametric curve described in a Cartesian system by a vector *v*(*s*), which is a function of a distance (*s*) along the curve^13,14^

(11) v(s)=[x(s),y(s),z(s)]

Assuming the vector *t* is a tangent vector tangent to a point trajectory. It can be concluded that the tangent vector *t* and the curvature vector of this point are

(12) $$\begin{array}{c} \left\{ \begin{array}{c} t=\frac{\mathrm{dv}}{\mathrm{ds}}\\ k=\frac{\mathrm{dt}}{\mathrm{ds}} \end{array}\right. \end{array}$$

Then, in the coordinate system, the curvatures projected on each of the XY plane, the YZ plane, and the XZ plane are

(13) $$\left\{ \begin{array}{c} k_{x}=\frac{dt_{x}}{\mathrm{ds}}\\ k_{y}=\frac{dt_{y}}{\mathrm{ds}}\\ k_{z}=\frac{dt_{z}}{\mathrm{ds}} \end{array}\right.$$

**Figure 2.**
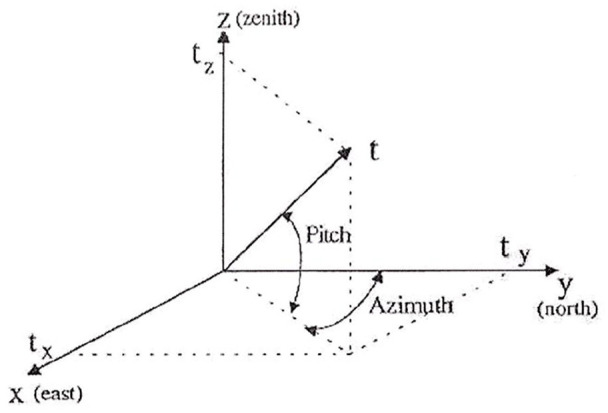
Pitch (*P*) and azimuth (*A*) for the pipeline centerline.

Assuming that the vector *t* is tangent of *v(s)*, separating the vertical and horizontal curvature components as shown in Figure 2

(14) $$\left\{ \begin{array}{c} t_{x}=\cos P\sin A\\ t_{y}=\cos P\cos A\\ t_{z}=\sin P \end{array}\right.$$

where the pitch (*P*) and azimuth (*A*) can be measured by the IIT for the pipeline centerline.

Assuming that the vector *k* is the curvature vector of a 3D curve at a given point, and the *k* consists of the vertical curvature $k_{v}$ and the horizontal curvature $k_{h}$ , which can be given as follows

(15) $$\left\{ \begin{array}{c} k(s)=\frac{\mathrm{dt}}{\mathrm{ds}}\\ k=\sqrt{k_{v}^{2}+k_{h}^{2}} \end{array}\right.$$

The above equation can be written separately for each component of the curvature vector in the Cartesian system

(16) $$\left\{ \begin{array}{c} k_{x}=\frac{dt_{x}}{\mathrm{ds}}\\ k_{y}=\frac{dt_{y}}{\mathrm{ds}}\\ k_{z}=\frac{dt_{z}}{\mathrm{ds}} \end{array}\right.$$

Based on equations (1)–(4), the components of the defined curvature vector can be calculated as follows

(17) $$\left\{ \begin{array}{c} k_{x}=-\sin P\left(\frac{\mathrm{dP}}{\mathrm{ds}}\right)\sin A+\cos P\cos A\left(\frac{\mathrm{dA}}{\mathrm{ds}}\right)\\ k_{y}=-\sin P\left(\frac{\mathrm{dP}}{\mathrm{ds}}\right)\cos A-\cos P\sin A\left(\frac{\mathrm{dA}}{\mathrm{ds}}\right)\\ k_{z}=\cos P\left(\frac{\mathrm{dP}}{\mathrm{ds}}\right) \end{array}\right.$$

The vertical curvature $k_{v}$ and the horizontal curvature $k_{h}$ can be given as follows

(18) $$\left\{ \begin{array}{c} k_{v}=-\frac{\mathrm{dP}}{\mathrm{ds}}\\ k_{h}=-\left(\frac{\mathrm{dA}}{\mathrm{ds}}\right)\cos P \end{array}\right.$$

The curvature *k* can be calculated as follows

(19) $$k=\sqrt{{\left(-\frac{1}{\sqrt{1-t_{z}^{2}}}\frac{dt_{z}}{\mathrm{ds}}\right)}^{2}+{\left(-\frac{1}{1+{\left(\frac{t_{x}}{t_{y}}\right)}^{2}}\left(\frac{1}{t_{y}}\frac{dt_{x}}{\mathrm{ds}}-\frac{t_{x}}{t_{y}^{2}}\frac{dt_{y}}{\mathrm{ds}}\right)\cos P\right)}^{2}}$$

According to the above formula and calculation, the formula for calculating the total and the vertical curvature $k_{v}$ and the horizontal curvature $k_{h}$ of the centerline of the pipeline are

(20) $$k=\sqrt{{\left(-\frac{1}{\sqrt{1-{\left(\frac{\mathrm{dz}}{\mathrm{ds}}\right)}^{2}}}\frac{d^{2}z}{ds^{2}}\right)}^{2}+{\left(-\frac{1}{\sqrt{1-{\left(\frac{\mathrm{dz}}{\mathrm{ds}}\right)}^{2}}}\left(\frac{\mathrm{dy}}{\mathrm{ds}}\frac{d^{2}x}{ds^{2}}-\frac{\mathrm{dx}}{\mathrm{ds}}\frac{d^{2}y}{ds^{2}}\right)\right)}^{2}}$$

(21) $$\left\{ \begin{array}{c} k_{v}=-\frac{1}{\sqrt{1-{\left(\frac{\mathrm{dz}}{\mathrm{ds}}\right)}^{2}}}\frac{d^{2}z}{ds^{2}}\\ k_{h}=-\frac{1}{\sqrt{1-{\left(\frac{\mathrm{dz}}{\mathrm{ds}}\right)}^{2}}}\left(\frac{\mathrm{dy}}{\mathrm{ds}}\frac{d^{2}x}{ds^{2}}-\frac{\mathrm{dx}}{\mathrm{ds}}\frac{d^{2}y}{ds^{2}}\right) \end{array}\right.$$

From equations (18) and (21), it can be seen that the pipeline bending strain is calculated with the trajectory of centerline and measured attitude of pipeline, which can be obtained from the IIT.

### Research on optimization for bending strain based on Symlets wavelet analysis

Based on equations (18) and (21), the calculation of pipeline bending strain is judged by IIT attitude or accurate trajectory. However, during the operation, the IIT is subjected to vibration and impaction due to the effects of girth welds, spiral welds, and so on. The calculation of the attitude data is disturbed. Thereby, it affects the calculation of pipeline bending strain.

In order to optimize the calculation of pipeline bending strain, the attitude information measured by IMU should be processed to eliminate system noise and other noise caused by girth welds, spiral welds, and so on. The wavelet analysis is a new type of time–frequency analysis method that has emerged in recent years.^15^ It has good localization properties in both time and frequency domains. The wavelet analysis can resolve the analyzed signals with multiple frequency components according to a certain time and frequency. It is effectively characterizing the abrupt characteristics of the signal.

Symlets wavelet is an orthogonal tightly supported wavelet function proposed by Ingrid Daubechies, which is improved by dbN wavelet.^16^ It is a discrete sequence wavelet transform based on multi-resolution analysis and multi-sampling rate filter theory. The Symlets wavelet is assumed as $m_{0}(\omega )=(1/2)\sum_{k=0}^{2N-1}h_{k}e^{-\mathrm{jk}\omega }$ , $|m_{0}(\omega ){|}^{2}$ is the function of $z=e^{\mathrm{jw}}$ which is defined as *W. W* can be analyzed as follows

(22) $$W(z)=U(z)\overset{---------}{U\left(\frac{1}{z}\right)}$$

**Figure 3.**
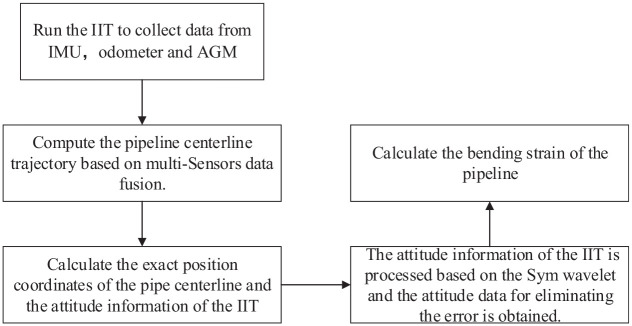
The flowchart for the optimization for pipeline bending strain.

Symlets wavelets are usually expressed as symN N=2,3,…,8 . The symN wavelet has a good regularity and its range is 2*N*– 1. Compared with the dbN wavelet, the Symlets wavelet is consistent with the dbN wavelet in terms of continuity, support length, and filter length, but the symN wavelet has better symmetry, that is, it can reduce the analysis and reconstruction of the signal to some extent. When we use discrete sequence wavelet transform to process discrete signal sequences, it is more suitable for fast calculation for pipeline in-line inspection. When choosing a wavelet function, factors such as its performance and filter length should be considered. Sym8 wavelet is used for optimization to the pipeline bending strain. The method of optimization for bending strain based on Symlets wavelet analysis is shown in Figure 3.

## Test and data analysis

### Characteristic for IIT and IMU

**Figure 4.**
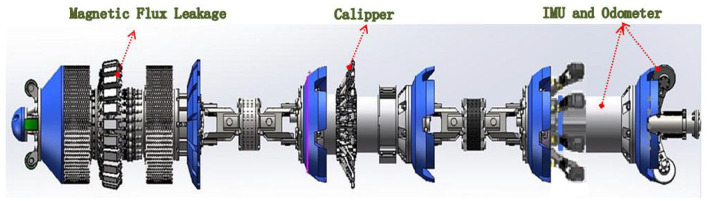
A typical 10″ integrated in-line inspection tool.

In order to inspect the small size pipeline, the special IIT is developed which installed with MEMS IMU, odometer, and low-frequency signal transmitter (or magnetic signal). In general, The IIT is integrated with other IIT such as MFL (magnetic flux leakage), caliper, and so on. A typical 10″ integrated IIT is shown in Figure 4. The characteristics of the sensors are shown in Table 1. The pipeline centerline measurements by accelerometers and gyros were used in the multi-sensors data fusion algorithm to estimate the position, velocity, and attitude of the IIT.

**Table 1.** Characteristic of sensors.

Sensor	Characteristics	Magnitude
Gyroscope	Bias	<3°/h
	Scaling factor	<50 ppm
Accelerometer	Bias stability	<20 ug
	Scaling factor	<80 ppm
Odometer	Scaling factor	<0.3%
	White noise	<0.1 m/s
AGM	White noise	<±1 m

AGM: above-ground marker.

### Data analysis for pull-through test

**Figure 5.**
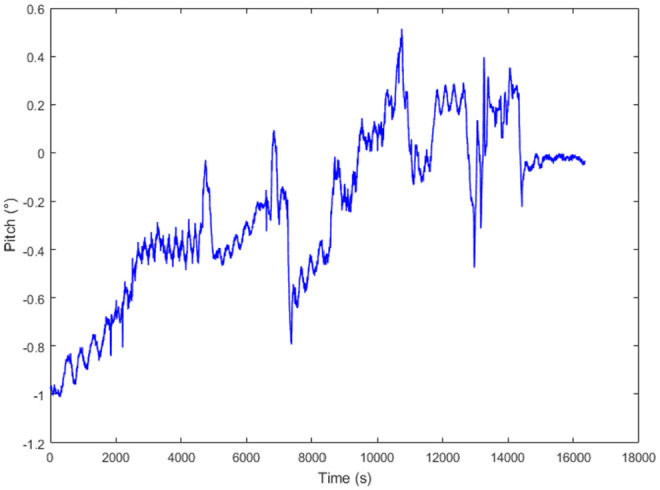
The attitude of the IIT for PHT.

**Figure 6.**
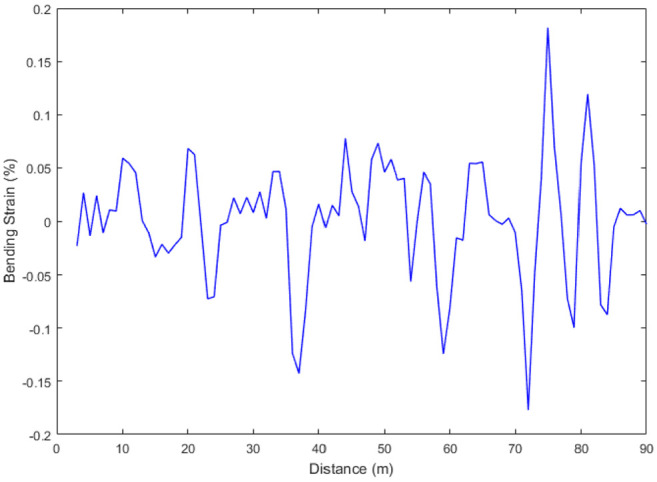
Calculation of bending strain of un-optimization for PHT.

The pull-through test (PHT) should be designed to verify the correctness of proposed method and the accuracy of inspection. In the test, 90-m straight pipeline is installed which mounted the strain gauge to compare the calculation with IIT. Data analysis for pipeline bending strain will be inspected and calculated for the straight pipeline and sank pipeline, respectively. Figures 5 and 6 show the attitude of the IIT and calculation of pipeline bending strain for PHT, respectively. Figure 5 shows that the IIT is affected by vibration, girth weld, and other feathers, which interferes the calculation of the bending strain.

**Figure 7.**
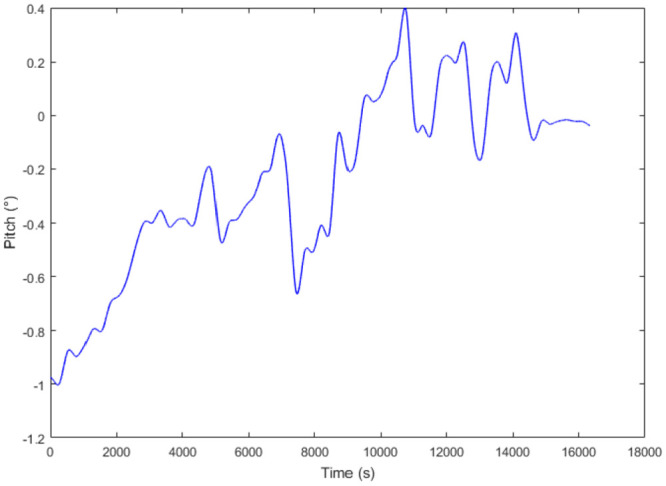
The attitude of the IIT for PHT after wavelet analysis.

**Figure 8.**
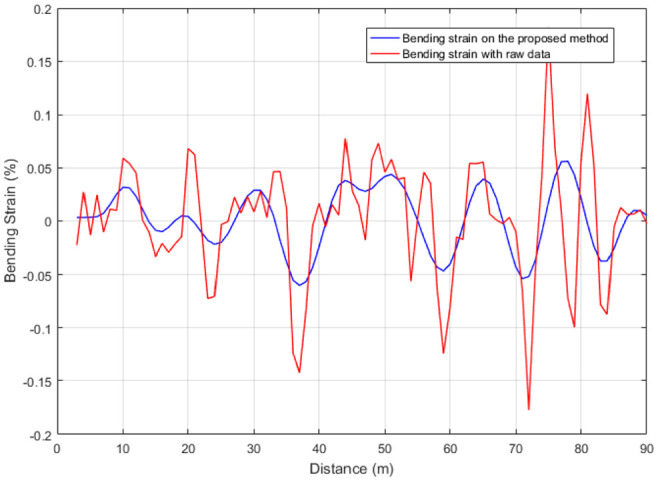
Comparison of bending strain for PHT.

According to proposed method, the attitude information is processed first. The attitude information is analyzed and de-noised by Sym8 wavelet in which the decomposition is at the eighth level. The results of attitude and pipeline bending strain are shown in Figures 7 and 8. It can be seen from the results that the noise interference of the fixed frequency due to the vibration of the IIT during the run is eliminated first. Second, due to the existence of girth welds and spiral welds in the pipeline, the IIT will be in vibration and shock when it passes these features. The IMU can sensitively collect these vibrations and shocks, so that the attitude data are disturbed by these noises. These noise disturbances will further affect the calculation of bending strain. After processing the attitude data by proposed method, it can effectively reduce the noise interference caused by these vibrations and shocks, and the calculation of bending strain is more accurate. In a further calculation, the pipeline bending strain is closer to the straight pipeline unstrained state. According to the field conditions, the calculated bending strain for the each girth weld is about ±0.05%, which provides an effective reference for the location of abnormal bending strain.

**Figure 9.**
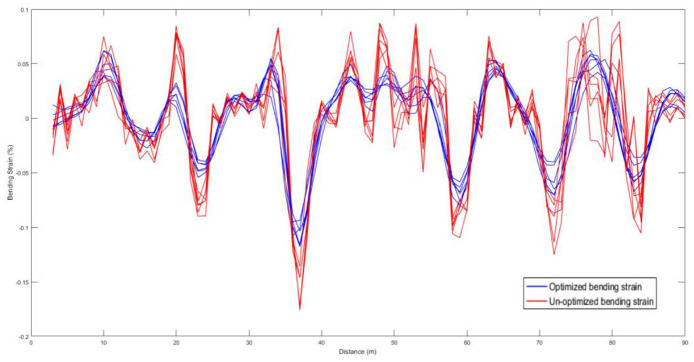
The comparison for un-optimized and optimized of bending strain.

In order to verify the validity and repeatability of the proposed method, six PHTs for straight pipeline have been carried out. The comparison for before and after processing of inspection and the calculation of bending strain are shown in Figure 9. From the results, it can be seen that the proposed method has good consistency and repeatability for the calculation of the bending strain of the pipeline body. Due to the unstable attitude at the girth weld, the bending strain deviation is slightly larger, and the maximum deviation is 0.03%. But it does not affect the analysis of the overall bending strain of the pipeline.

In order to verify the accuracy of changes of the bending strain for the proposed method, the strain gauge is mounted to the position of the pipeline strain maximum changes for pipeline sink. The strain gauge is used to measure the strain change generated by the pipeline offset, and then to compare the result with the calculation of IIT and strain gauge measurement.

**Figure 10.**
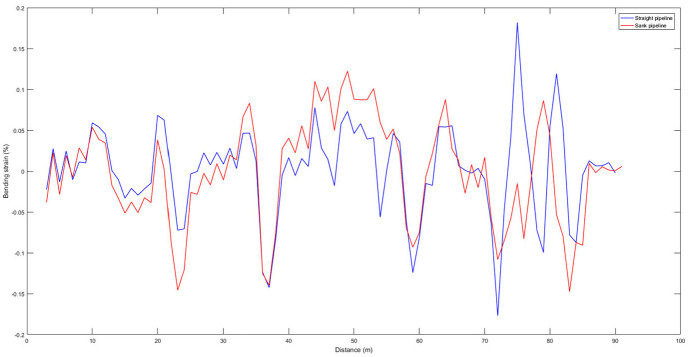
The comparison of un-optimized bending strain for sank pipeline.

The pipeline is sank 25.5 cm with loadings in which the maximum changes of the pipeline strain is at the middle of the pipeline. Strain gauge is mounted at 47 m to measure pipeline strain changes. These strain gauges should be avoided to mount at the girth weld. The comparison of un-optimized bending strain for straight and sank pipeline is shown in Figure 10.

**Figure 11.**
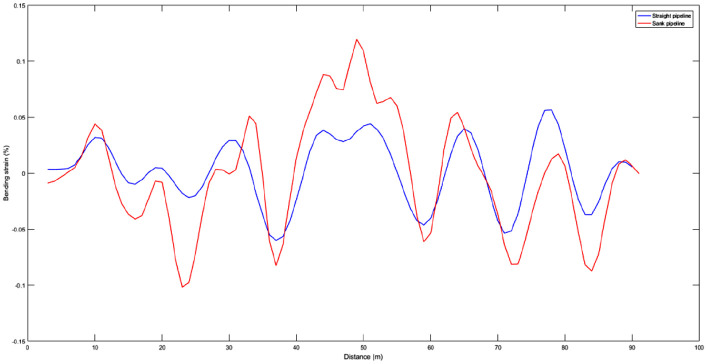
The comparison of optimized bending strain for sank pipeline.

The comparison of bending strain for straight and sank pipeline with proposed method is shown in Figure 11, and the synthesized comparison is shown in Table 2. The measurement of the strain changes for strain gauge is 0.04926%. The calculation of un-optimized pipeline bending strain for IIT is 0.062%, and absolute deviation and relative deviation with strain gauge are 0.013% and 25.8%, respectively. The optimized calculation for IIT is 0.046%, and absolute deviation and relative deviation with strain gauge are 0.003% and 6.7%, respectively. At present, some papers show results providing absolute deviation for bending strain is 0.005% to 0.01% based on the method of moving average or low-pass filtering. Some specifications or standards specify the absolute deviation is 0.02%. It can be seen from the results that the optimized calculation of the pipeline bending strain is more accurate, which can effectively measure and evaluate the bending strain of the pipeline after deformation. The proposed method provides an effective method for bending strain in-line inspection of long-distance and small-size pipelines.

**Table 2.** The comparison for of bending strain for sank pipeline.

Position of strain gauge	Strain changes for gauge	Un-optimized bending strain			Optimized bending strain		
		Strain changes	Absolute deviation	Relative deviation	Strain changes	Absolute deviation	Relative deviation
47 m	0.04926%	0.062%	0.013%	25.8%	0.046%	0.003%	6.7%

## Conclusion

In order to inspect the stress and strain of oil and gas pipelines in geological disaster areas, the in-line inspection of pipeline bending strain uses high-end tactical-grade IMU which has become the routine practice. But for the small-size pipeline, the MEMS IMU is use to inspect the pipeline bending strain. Due to the large error of the MEMS inertial navigation system, it is easy to receive external disturbances during operation such as the girth weld and vibration. The trajectory and attitude information of the IIT are disturbed, which affects the accuracy of the inspection. The trajectory and attitude information of the IIT are affected during accuracy of the inspection, and the accuracy of pipeline bending strain is decreased during the inspection. This article proposes the method to calculate and optimize the pipeline bending strain. It can be obtained that

1. This article provides the algorithm for trajectory of small size pipeline centerline based on multi-sensors data fusion and the calculation method of the pipeline bending strain.
2. A method to optimize and improve the accuracy for the bending strain based on the wavelet analysis is proposed.
3. The effectiveness of the method was verified by repeatability and displacement PHT, and it will increase the accuracy to within 19.1% of the actual bending strain.

As a result of geological disasters or third-party damage, small-size oil and gas pipelines are more likely to bend due to external forces, and it is easy to become high-risk segments because of local stress concentration. Through accurate in-line inspection based on MEMS IMU of pipeline bending strain, combined with caliper or MFL inspection of pipeline defects, it is possible to accurately identify and locate high-risk segments that pose a potential threat to the safety operation of the pipeline, to make targeted monitoring and repair plans. It is significant to effectively guarantee the essential safety of small-size oil and gas pipelines.
